# Myocardial Edema without Fibrosis by Magnetic Resonance T2 Mapping in Acute Chagas’ Myocarditis

**DOI:** 10.5935/abc.20170113

**Published:** 2017-10

**Authors:** Andréa Silvestre de Sousa, Maria Eduarda Derenne, Alejandro Marcel Hasslocher-Moreno, Sérgio Salles Xavier, Ilan Gottlieb

**Affiliations:** 1 Instituto Nacional de Infectologia Evandro Chagas - Fundação Oswaldo Cruz, Rio de Janeiro, RJ - Brazil; 2 Hospital Universitário Clementino Fraga Filho - Universidade Federal do Rio de Janeiro (UFRJ), Rio de Janeiro, RJ - Brazil; 3 Casa de Saúde São José, Rio de Janeiro, RJ - Brazil; 4 Instituto Nacional de Cardiologia, Rio de Janeiro, RJ - Brazil

**Keywords:** Acute Chagas’ Myocarditis, Parametric Mapping

**Figure 1:**
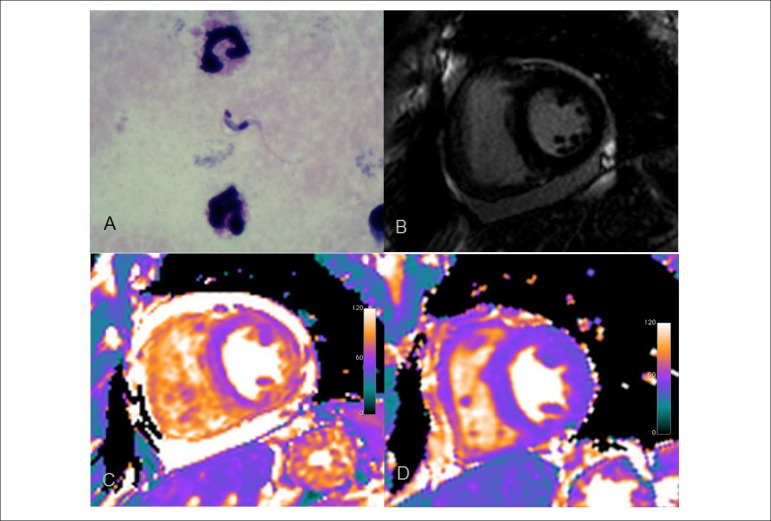
Trypanosoma cruzi parasite at thick blood smear in acute Chagas disease (A); first cardiac magnetic resonance with no myocardial fibrosis at late gadolinium enhancement (B), but with moderate pericardial effusion and myocardial T2 times of 70-72 ms compatible with edema in all myocardial segments (C); complete edema regression (T2 = 45-48 ms) and no pericardial effusion after specific treatment (D).

A 47-year old previously healthy male presented fever and malaise for 30 days. Chagas’ disease was diagnosed by direct visualization of *Trypanosoma cruzi* parasites at thick blood smear (Figure 1A). Benznidazole was started and symptoms gradually subsided. At presentation, the patient had low QRS voltage and primary repolarization abnormalities on ECG, normal troponin level, moderate pericardial effusion and normal systolic function of both ventricles on echocardiogram. Cardiac magnetic resonance (CMR) using a 3T system (Verio, Siemens Healthcare) was performed five days after the treatment started and confirmed normal biventricular function and cavity sizes and moderate pericardial effusion. Late gadolinium enhancement (LGE) was normal (Figure 1B), but parametric T2 mapping of the myocardium (Siemens Healthcare) revealed myocardial T2 times of 70-72 ms (normal < 50 ms) compatible with edema in all myocardial segments (Figure 1C). A second CMR study, 26 days after treatment initiation, showed no pericardial effusion and partial regression of myocardial edema with T2 times of 50-54ms. A third study, 56 days after treatment initiation, showed complete regression of myocardial edema, with T2 times of 45-48 ms (Figure 1D). LGE was always negative. Direct detection of the parasite in the bloodstream was negative 13 days after treatment. This well documented acute Chagas’ myocarditis case had no myocardial fibrosis. Nonetheless, exuberant myocardial edema was present that gradually subsided 56 days after specific treatment was started. T2 mapping was able to identify myocardial involvement beyond conventional CMR techniques as LGE, and it was demonstrated for the first time for acute Chagas disease.

